# Multiple Myeloma: Real‐world Data on the Clinical Presentation and Outcomes From Oman

**DOI:** 10.1002/jha2.70096

**Published:** 2025-07-21

**Authors:** Ibrahim Al Nabhani, Jaber Al Lawati, Nooh Al Mahrooqi, Adnan Al Rawahi, Vinodh Kumar, Nafila Al Riyami, Khalid Al Waili, Zeba Jabeen, Arwa Z. Al‐Riyami, Hammad Khan, Mohammed Al Huneini, Thuraya Al Busaidi, Salam Al Kindi, Murtadha Al Khabori, Mahmood Al Abri, Najla Fawaz, Khalil Al Farsi

**Affiliations:** ^1^ Department of Hematology Sultan Qaboos University Hospital Muscat Oman; ^2^ Department of Medicine Royal Hospital Muscat Oman; ^3^ Department of Biochemistry Sultan Qaboos University Hospital Muscat Oman

**Keywords:** light chain | multiple myeloma | Oman | SQUH

## Abstract

**Objective:**

There is a paucity of data on multiple myeloma (MM) from Oman and the region. This study reports the clinical presentation and survival outcomes in Omani patients with MM at Sultan Qaboos University Hospital (SQUH), a tertiary care academic center.

**Methods:**

This retrospective included all patients diagnosed, treated, and followed up for MM at SQUH between June 2008 and December 2018. Patient demographics, disease characteristics, clinical presentation, prognostic parameters, and survival data were obtained from the patients’ electronic medical records. The Kaplan‐Meier method was used to estimate the progression‐free survival (PFS) and overall survival (OS), and the log‐rank test was used to compare survival according to the international staging system (ISS) stage.

**Results:**

Ninety‐eight patients were analyzed, 49 (50%) of whom were males. The median age was 61 years (range: 30–88). Immunoglobulin G (IgG) was the most common subtype of myeloma (59.6%), followed by IgA (24.2%) and IgD (4%). Twelve patients (12%) had light chain myeloma. The most common manifestation of myeloma at the time of initial diagnosis was anemia (50%), followed by lytic lesions (41.5%), renal insufficiency (24.2%), and hypercalcemia (23.2%). Forty‐three patients (44%) had ISS stage III disease at presentation. Over the study period, patients received different types of induction therapy. Ninety patients received therapy at SQUH and had complete data on first‐line treatment. Thirty‐six patients (41%) received proteasome inhibitor (PI) based regimens, 23 patients (25%) had immunomodulatory‐based (IMID‐based) therapy, and 23 patients (25%) had combination PI and IMiD‐based induction therapy. For patients with complete data on treatment, responses and outcome (*n* = 90), after a median follow‐up of 76 months (95% confidence interval [CI]: 54–97 m), median PFS was 59 months (95% CI: 20.1–78), with an estimated five year PFS of 41%. Median OS was 109 months (95% CI: 65–173) with a 5‐year OS of 61%. ISS stage predicted OS (ISS stage I and stage II: 133 m, 95% CI: 94–173 m, stage III: 36 m, 95% CI: 25–47 m; log‐rank *p* < 0.001) but not PFS.

**Conclusion:**

The median age of our patients is younger than what is published in the literature. Most of our patients presented with advanced‐stage disease, which was predictive of survival in our cohort. The lack of uniformity of treatment and the small number of patients precluded concluding the effect of treatment on survival. Collaboration with other centers in Oman and the region to collect retrospective and prospective data on a larger cohort of patients is recommended.

**Clinical Trial Registration:**

The authors have confirmed that clinical trial registration is not needed for this submission.

## 1 Introduction

Multiple myeloma (MM) is characterized by the proliferation of malignant plasma cells (≥ 10% clonal plasma cells in the bone marrow or biopsy‐proven plasmacytoma) that produce monoclonal immunoglobulins and/or free light chains and cause one or more of the myeloma‐defining events. These include hypercalcemia (serum calcium > 2.75 mmol/L), renal failure (serum creatinine >177 mol/L), anemia (hemoglobin ≤ 10 g/dL), and bone lytic lesions, collectively referred to as CRAB. In addition, the presence of ≥ 60% plasma cells in the bone marrow, a serum‐free light chain ratio of ≥100, or more than one focal bone lesion on magnetic resonance is also considered diagnostic of symptomatic myeloma [1, 2]. MM accounts for about 1% of all cancers and 10% of hematological malignancies in the United States [3]. The exact incidence in Oman is unknown. Myeloma is a disease of older patients with a median age at diagnosis between 65 and 75 years [4]. Staging and prognosis are determined by several factors that are now part of the revised international staging system (R‐ISS). These include serum albumin, serum β_2_ macroglobulin, serum lactate dehydrogenase (LDH), and cytogenetic abnormalities (of which 17p deletion, t(4;14) and t(14;16) are considered high risk). Additional high‐risk cytogenetic abnormalities include t(14;20), gain of chromosome 1q, and any alterations in the p53 gene on chromosome 17 [5].

Although the disease remains incurable, current treatment strategies have significantly improved patient survival [6, 7]. Typically, younger (≤65 years of age) fit patients receive induction therapy followed by high‐dose chemotherapy and autologous stem cell transplant, followed by maintenance therapy until disease progression. Older and less fit patients, on the other hand, receive prolonged induction therapy followed by continuous maintenance. The use of combinations of novel agents, including bortezomib (V), lenalidomide (R), and/or others, along with dexamethasone (D), has led to long survival times. The combination VRD, for example, has led to median progression‐free (PFS) and overall survivals (OS) over 70 months [8]. More recent data on the use of daratumumab in combination with one or more of these agents has led to further improvement in survival [9, 10, 11].

There are no available data on the presentation and outcome of patients with MM from Oman, and there is very limited data from the region. Our objectives are to describe the clinical and laboratory characteristics at diagnosis, report on the treatments offered, and analyze the survival outcomes of Omani patients with MM treated at SQUH.

## 2 Methods

This is a retrospective chart review of patient health records at SQUH. It includes all Omani patients with MM who were diagnosed, treated, and had complete follow‐up data at SQUH, a tertiary care academic referral hospital affiliated with the College of Medicine and Health Sciences (COM&HS) at Sultan Qaboos University in Muscat, between June 2008 and December 2018. Data collected include demographic data (age and gender), disease‐specific variables, including findings from baseline laboratory tests (hemoglobin, calcium, creatinine, albumin, B2 macroglobulin), imaging studies, and cytogenetic analyses. Treatment and outcome data were also collected. The primary outcome is PFS, defined as the time from the diagnosis of MM until the first sign of biochemical and/or clinical progression, relapse, last follow‐up, or death. The secondary outcome is OS, defined as the time from diagnosis to the time of death or last follow‐up.

The sample size was calculated based on the assumption of a 2‐year PFS of 65% and is based on sample size calculation for proportions with finite populations, a precision of 10%, power of 80%, and a *p*‐value of 0.05 as significant, with a confidence interval (CI) of 95%. The minimum sample size required was 88. Categorical data are reported here as frequencies and percentages. Continuous data are presented as means/medians (with standard deviations) as appropriate. The Kaplan‐Meier method was used to estimate the median PFS and median OS. The log‐rank test compared survival differences according to the ISS stage. A *p*‐value of < 0.05 was considered significant. The analysis was done using the statistical software SPSS Statistics 23 (SPSS; IBM Corp., Armonk, NY, USA). Ethical approval was obtained from the COM&HS Ethical Committee.

## 3 Results

There was a total of 98 patients were analyzed. Table 1 summarizes the patient population's primary demographic, clinical, and treatment characteristics. Median age was 61 years (range: 30–88 years). Sixty‐six patients (67%) were 65 years or younger (all of whom were transplant‐eligible and received autologous transplants following induction therapy). 50% of patients were males. IgG subtype was the most common subtype of myeloma (about 60%), followed by IgA (24%), free light chain myeloma (12%), and IgD (4%). Anemia was present in half of the patients, lytic lesions in 42%, renal insufficiency in 25% and hypercalcemia in 24% at diagnosis. Thirty‐nine patients (44%) had β_2_ macroglobulin levels > 5.5 mg/L, and 44 patients (49%) had serum albumin less than 35 g/L. The median LDH level was 186 U/L (SD: 408, range: 73–3447), with 25 patients (28%) having high LDH. Of 90 patients with complete data on the ISS stage, first‐line treatment, and follow‐up, 46 (51%) had stage III disease at presentation, while 44 patients (49%) were at stages I and II. Data on fluorescence in situ hybridization (FISH) at diagnosis were available only for 24 patients. Five patients had t(4;14), one had t(14;16), two had 17p deletion, and one had 1q gain, while trisomies were seen in four patients and t(11;14) in 3 patients. Out of these 24 patients, three patients had two or more poor prognostic cytogenetic alterations at diagnosis.

**TABLE 1:** Baseline clinical and treatment characteristics of the study population.

Characteristic	Number	Percentage
Median age	61 (range: 30–88)	
Age ≤ 65 years	66	67%
Age > 65 years	32	33%
Male	49	50%
MM subtype		
•IgG	59	60%
• IgA	23	24%
• IgD	4	4%
• FLC	12	12%
Clinical presentation		
• Anemia	49	50%
• Renal insufficiency	24	24%
• Hypercalcemia	23	24%
• Lytic lesions	41	42%
ISS Stage (*n* = 90)		
• Stage 1–2	46	51%
• Stage 3	44	49%
Treatment (*n* = 90)		
• PI based^a^	37	41.1%
• IMiD based^b^	23	25.6%
• Combination PI & IMiD	23	25.6%
• Others	7	7.8%

^a^ PI‐based: proteasome inhibitor therapy.

^b^ IMiD‐based: immunomodulatory‐based therapy.

## 4 Treatment and Outcome

Thirty‐seven patients (41%) received proteasome inhibitor (PI)‐based regimens; bortezomib‐dexamethasone (Vd) in 16 of the total 90 patients (18%), bortezomib‐cyclophosphamide‐dexamethasone (VCd) in 13 (14%), bortezomib‐melphalan‐dexamethasone (VMP) in seven (8%), and one patient who was not able to receive R had daratumumab‐bortezomib‐dexamethasone (DVD). Twenty‐three patients (25.6%) had immunomodulatory‐based (IMID‐based) therapy; 15 (16%) thalidomide‐dexamethasone (TD), six (7%) lenalidomide‐dexamethasone (Rd), and two had melphalan‐prednisone‐thalidomide combination (MPT). Twenty‐three patients (25.6%) had combination PI and IMiD‐based induction therapy; 21 (23%) had bortezomib‐lenalidomide‐dexamethasone (VRd), one had bortezomib‐thalidomide‐dexamethasone (VTd), and one had carfilzomib‐lenalidomide‐dexamethasone (KRd).

**FIGURE 1:**
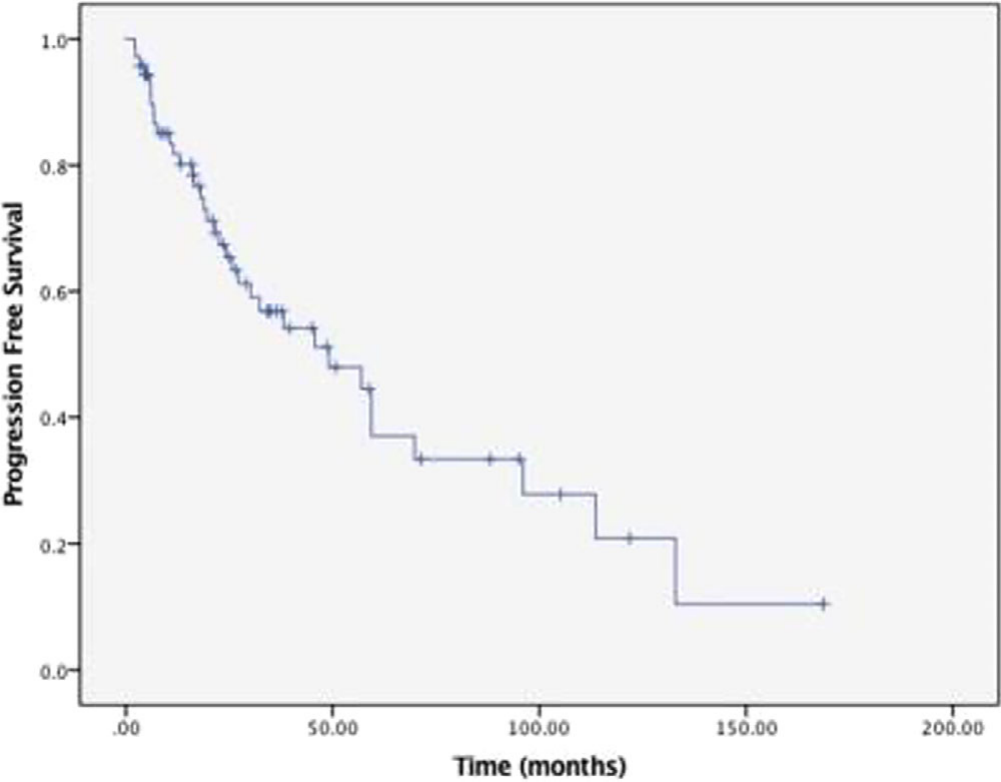
Progression‐free survival (PFS) curve for the overall group of 90 patients who had complete data on treatment, responses, and follow‐up.

**FIGURE 2:**
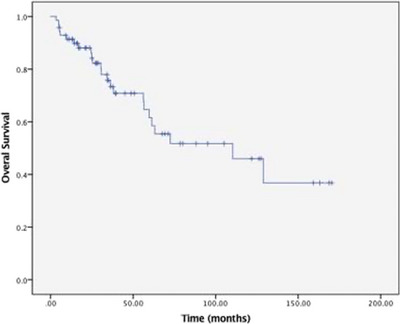
Overall survival (OS) curves for the overall group of 90 patients who had complete data on treatment, responses, and follow‐up.

**FIGURE 3:**
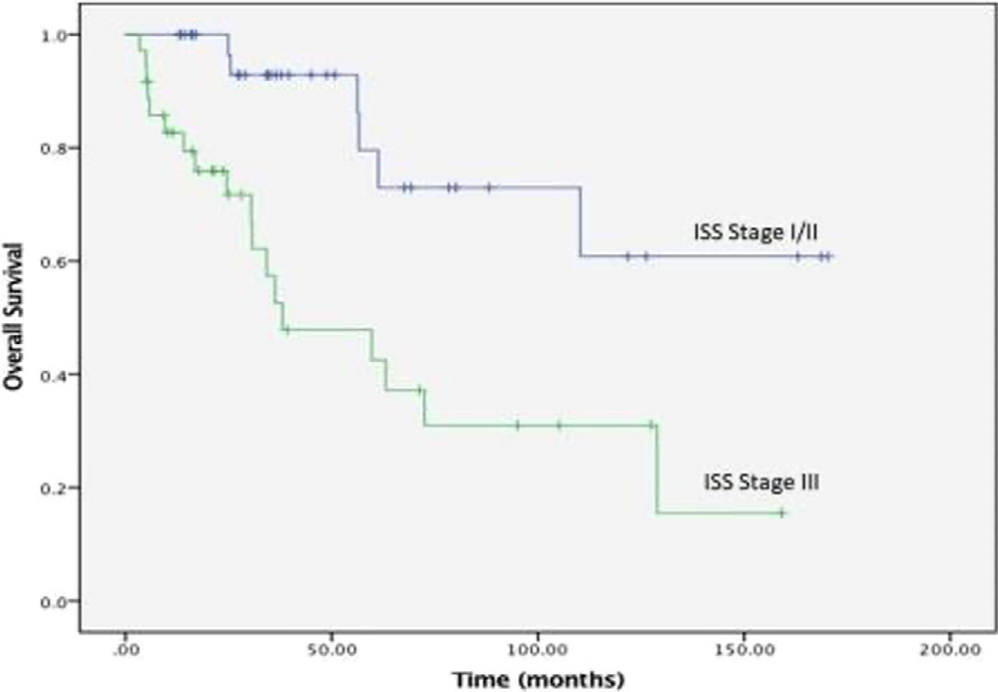
Overall survival (OS) curve as per international staging system (ISS) Stage (ISS Stage III at diagnosis was associated with inferior OS compared to stage I/II).

Figures 1 and 2 show the PFS and OS survival curves for the overall group of 90 patients who had complete data on treatment, responses, and follow‐up. With a median follow‐up of 76 months (95% CI: 54 – 97 months), the median overall PFS was 59 months (95% CI: 47–70 months) with an estimated 5‐year PFS of 41%. For patients 65 years and younger, the median PFS was 57 months (95%CI 46–68 months) with a 5‐year PFS of 42%. In patients over 65 years, median PFS was 59 months (95% CI 33–85 months), with a 5‐year PFS of 34%. The median PFS for stage III patients was 57 months (95% CI: 14–100 months) compared to 59 months (95% CI: 47–71 months) for stage I and stage II, with a log‐rank *p*‐value of 0.985. The median OS was 109 months (95% CI: 65–152 months), with an estimated 5‐year OS of 61% for the whole population. The median OS for patients 65 years and older was 110 months (95%CI: 57–163 months) with a 5‐year OS of 64%. For those over 65, the median OS was 61 months (95%CI: 65–152 months) with a 5‐year OS of 56%. ISS Stage III at diagnosis was associated with inferior OS compared to stage I/II (Figure 3). The median OS for stage III patients was 36 months (95% CI 25–47 months) compared to 133 months (95% CI 94–173 months) for stage I/ II, log‐rank *p*‐value <0.001, hazard ratio 3.1 (95% CI: 1.63–5.95). There was no significant difference in PFS and OS with the type of induction therapy (VRD vs. others), *p* = 0.19.

## 5 Discussion

This single‐institution retrospective study is the first report on MM from the country and one of the very few from the region. Our results from the analysis of this ethnically homogeneous patient population show that the median age of our patients (61 years) is lower than what is reported in the literature [4]. Reports from the region are limited. A report from Saudi Arabia on the outcome of autologous transplant for myeloma patients reported a median age of 51 years [12], while another report of 36 patients reported a median age of 65 years [13]. Two reports from the United Arab Emirates gave different estimates; one said a median age of 43 years [14], while the other reported a median age of 58 years [15]. A more extensive study of 217 patients from Egypt reported a similar median age (58 years). [16]

The subtypes of myeloma and the major clinical presentations are similar to what is reported in the literature [4] except for bone lytic lesions, which are seen in about half of our patients at diagnosis, compared to 20%–25% in other series. This is probably a reflection of the imaging modality used since our study included patients from more recent years where positron emission tomography‐computed tomography (PET‐CT) scan, magnetic resonance imaging (MRI), and low‐dose whole body CT scan are used, and it is not unexpected to see such higher rates. Anemia was our patients' most common laboratory finding (50%). Hypercalcemia and renal insufficiency are seen at a similar rate, about 25%. Advanced‐stage disease (ISS stage III) was seen in nearly half of our patient population, which is higher than what is reported in Western countries. [17] This can be explained by late diagnosis or late referrals. Alternatively, our patients may have a different disease biology, as a significant proportion of patients who had FISH done at diagnosis had high‐risk cytogenetic features.

Our current standard induction therapy for myeloma is VRd based on the results of the SWOG 0777 trial, which reported a median PFS of 43 months and median OS of 75 months [8]. About 25% of our patients received a combination of a PI, an IMiD, and D (mostly VRd; 21 out of the 90 patients). However, the rest received a variety of other induction regimens. VCd (CyBorD) was the second most common treatment regimen. Daratumumab, which has become one of the standards of care in myeloma, was not widely used in induction at our institution during this period, and only one patient in this cohort received daratumumab‐based induction. Data on overall response rates and depth of response following induction therapy are being collected. In such a retrospective study, safety data collection is challenging, but data on significant adverse events, including peripheral neuropathy, thrombosis, and infections, are being collected.

The median PFS in our patients (PFS 59 months), with a long follow‐up duration of over six years, is longer than that reported from nearby Gulf countries [12, 18]. It is also longer than seen in the SWOG0777 trial mentioned above (median of 43 months) [8]. The median OS was not reached in the SWOG 0777 after a median follow‐up of 84 months [19], while ours was 109 months with a median follow‐up of 76 months. 5‐year OS in the SWOG 0777 trial was 69% in those treated with VRd compared to 61% for the whole cohort in our study. This is not a matched comparison, and our patients received different types of induction therapies, with a minority receiving VRd or Rd. The difference in PFS can be explained by mainly including younger, fit patients in this analysis. In addition, the issue of this being a single institution study, with potential risks of referral and selection bias, may account for some of the differences. Differences in disease biology cannot be excluded. This result is interesting, given the impression amongst practicing hematologists in the country and the region that our patients do not do as well as those reported in the Western literature. The likely difference in OS (between our cohort and those in SWOG 0777) is expected due to the availability of a wide range of novel agents beyond first‐line therapy for patients progressing in the SWOG study. A more extensive collaborative study (with a correlative essential scientific part) with a matched comparison to patients from the US or Europe is needed. Another important finding from our research is that it confirms the adverse prognostic impact of the advanced ISS stage on survival in our patients. A statistically significant difference in OS according to disease stage was noted. We combined stages 1 and 2 because of the small number of these patients compared to patients with stage III. Compared to stage I/II, stage III disease had a significantly lower median OS with a hazard ratio of 3.1, as shown in Figure 3.

We acknowledge the limitations of our study, including its retrospective nature and relatively small sample size. This single institutional study may be subject to referral and selection biases. Results cannot be generalized to other countries or regional centers. Data on cytogenetics and FISH were not available for many patients. As a result, the revised ISS stage (now used as the staging and prognostic system in myeloma) could not be calculated, and its impact on survival in our patients could not be analyzed. We used the ISS stages instead and were able to validate their prognostic impact on survival. With such a long study period, patients had different induction regimens over the years. Supportive care has improved over the study period. Analysis of more recent cohorts of patients may produce different results. The findings that the type of treatment has no significant impact on PFS and OS need further confirmation because of the relatively small number of patients receiving each of these regimens. Data on additional patients treated with VRd in the past few years are being collected.

Despite these limitations, this study is the first from the country and is one of the most extensive and detailed from the region. The duration of follow‐up is one of the longest reported in the literature. This study will provide a platform for future research, including collaborative research with other centers from the country, region, and the rest of the world. It would also serve as the nucleus for a national (and regional) database on myeloma and other related disorders that could be used for further research and may be utilized in planning the health care budget.

## 6 Conclusion

Our study, the first on myeloma from Oman, showed a younger patient population with more favourable survival outcomes despite advanced‐stage disease in half of the patients. Results must be confirmed in more extensive multicenter collaborative studies from the country and the region. Prospective real‐world data with attention to disease biology, treatment type, efficacy, safety, patient quality of life, and economic impact is needed.

## Conflicts of Interest

The authors declare no conflicts of interest.

## Data Availability

The datasets collected and/or analyzed during the current study are not publicly available due to institutional restrictions/patient confidentiality/ethical approval limitations, but are available from the corresponding author on reasonable request.
